# The profile of patients with haemophilia managed at a haemophilia treatment centre in Pretoria, Gauteng

**DOI:** 10.4102/safp.v64i1.5551

**Published:** 2022-10-13

**Authors:** Lethukuthula Mafisa, Abegail N. Dlova, Vanessa Moodley

**Affiliations:** 1 Department of Haematological Pathology, School of Medicine, Sefako Makgatho Health Sciences University, Pretoria, South Africa; 2 Department of Haematological Pathology, Dr George Mukhari Academic Laboratory, National Health Laboratory Service, Pretoria, South Africa

**Keywords:** haemophilia, home therapy, prophylaxis, comprehensive management, inhibitors, coagulation factor deficiency

## Abstract

**Background:**

Haemophilia A and B are X-linked recessive bleeding disorders resulting from a deficiency of factors VIII and IX, respectively. Early diagnosis and a comprehensive approach to management is mandatory. This study aimed to describe the profile of patients with haemophilia (PWH) managed at Dr George Mukhari Academic Hospital (DGMAH) with the view to identify potential areas to improve haemophilia care.

**Methods:**

A cross-sectional, descriptive study that retrospectively reviewed clinical and laboratory records of PWH managed at DGMAH haemophilia treatment centre from 01 January 2003 to 31 December 2017.

**Results:**

Forty-four males were identified, with the majority being adults (~61%). Haemophilia A patients (~82%) outnumbered those with haemophilia B (~18%). Spontaneous mucocutaneous bleeding was the most frequent presenting feature followed by haemarthrosis. Disease-related complications included joint complications and life-threatening bleeds. There was a delay in diagnosis in 11% PWH. Management included episodic plasma-derived factor replacement and bypassing agents for patients with inhibitors. Only 13% of PWH were on home therapy. Prevalence of inhibitor development was 18%. There was a paucity of recorded data regarding prophylaxis, genetic counselling, psychological and physiotherapy support.

**Conclusion:**

The majority of PWH were adults, and haemophilia A was more prevalent than haemophilia B. A delay in haemophilia diagnosis could be addressed by increasing the awareness of haemophilia in health facilities. Expanding home therapy and introducing prophylaxis will likely improve the quality of life in PWH. Study outputs have included compilation of diagnostic and management algorithms to optimise haemophilia care at DGMAH.

## Introduction

Haemophilia is a life long bleeding disorder caused by a deficiency of coagulation factors VIII, IX or XI resulting in haemophilia A, B or C, respectively.^1,2^ Both haemophilia A (HA) and haemophilia B (HB) are inherited in an X-linked fashion, whereas the mode of inheritance of haemophilia C (HC) is autosomal recessive, seen mostly in Ashkenazi Jews; therefore HC will not be discussed further.^2^ Haemophilia A and haemophilia B can also be acquired through immunological means.^3^ Globally, the prevalence of HA and HB is reported to be 1:5000 and 1:30 000 male births, respectively.^4^ The severity in bleeding phenotype is reported to be similar in severe HA and HB, with spontaneous joint and muscle bleeds being more frequent.^5,6^ The onset of bleeding is typically early, from six to eight months of age, as physical activity increases.^7^ A delay in diagnosis has been reported in patients with haemophilia (PWH) in African countries including South Africa (SA) and Cameroon. This was suggested to be because of a lack of awareness of the disease.^8,9^ The provision of comprehensive haemophilia care has been hindered by the availability of resources in some countries. The study by Payal et al. performed in India cites limited resources as a hindrance to providing comprehensive haemophilia care.^10^ In the said study, patients received episodic treatment, with clotting factor replacement issued to only 45% of the patients, whilst the rest of the patients received cryoprecipitate and fresh frozen plasma.

The World Federation of Haemophilia (WFH) guidelines advocate for a comprehensive and multidisciplinary approach for the management of PWH.^11^ Factor replacement therapy (FRT) is the mainstay of therapy worldwide, with prophylaxis reported to be superior to episodic treatment.^11,12,13^ Prophylaxis is defined as the regular administration of a haemostatic agents in PWH to improve haemostasis and effectively prevent bleeding.^11^ Primary prophylaxis is the regular, continuous prophylaxis in the absence of joint disease, documented clinically and/or by imaging and before the second clinically evident joint bleed and before three years of age.^11,13^ The SA treatment guidelines recommend primary prophylaxis for infants with severe haemophilia, as they have an increased risk of developing haemophilic arthropathy.^14^

Secondary prophylaxis is the regular continuous prophylaxis commenced after two or more joint bleeds but before there is detectable clinical and/or radiological joint disease.^11^ In SA, secondary prophylaxis is to be considered for managing chronic synovitis and may be given intermittently prior to activities that may result in bleeding. Tertiary prophylaxis is the regular, continuous prophylaxis in patients who have confirmed clinical and/or radiological joint disease.^11^ Its aim is to prevent disease progression.^11^

Prophylaxis is the mainstay of treatment in well-resourced countries, and it has been associated with an improvement in the quality of life in PWH.^15^ Developing countries face several challenges with regard to haemophilia care. Besides the delay in diagnosis, limited access to treatment results in widespread joint deformities with high morbidity.^10^ The aim of this study was to determine the profile of PWH at Dr George Mukhari Academic Hospital (DGMAH), with a view to optimise the care of these patients.

## Material and methods

This was a cross-sectional, descriptive study with retrospective clinical and laboratory record review. The study population included all patients with a confirmed diagnosis of haemophilia who attended the haemophilia treatment centre (HTC) at DGMAH from 01 January 2003 to 31 December 2017. There were no exclusion criteria. A total of 44 patients with a diagnosis of congenital haemophilia and a single patient with acquired HA were identified, with the latter discussed separately.

Patient data included clinical information such as sex, age, type of bleed, bleeding site, family history of haemophilia, management and complications including disease (life-threatening bleeds and musculoskeletal, including target joints) and treatment-related (inhibitor development). In addition, laboratory test results (factor assays, viral studies, genetic studies and inhibitor assays) were captured. Target joints are defined as three or more joint bleeds occurring spontaneously into a single joint within a consecutive 6-month period. Life-threatening bleeding (LTB) is defined as severe bleeds requiring urgent treatment because of high risk of immediate death. Diagnostic delay was considered in this study to be the difference in time from the onset of first bleeding to the time of diagnosis.

Factor VIII and IX levels were measured using a one-stage activated partial thromboplastin time (APTT) based assay on an automated analyser. Haemophilia is graded according to the International Society for Thrombosis and Haemostasis as severe if the factor level is less than 1% (< 1 IU/dL), moderate if it is between 1% and 5% (1 IU/dL – 5 IU/dL) and mild if it is greater than 5% but less than 40% (> 5 < 40 IU/dL).^11,14^ Activated partial thromboplastin time mixing studies were performed as a screening test to detect the presence of inhibitors at 6-monthly intervals as per SA guidelines. If the inhibitor screen was positive, the Bethesda assay was performed to determine the inhibitor titre.

Demographic details were summarised descriptively using frequency tables and graphs. Frequency tables were constructed for clinical data, laboratory results and management. Pearson correlation and Pearson chi-square tests were performed to associate severity and clinical complications and severity and immunological complications. A *p*-value of ≤ 0.05 was used to determine statistical significance. All statistical procedures were performed on IBM Statistical Package for Social Sciences (SPSS) Statistics 24, running under Microsoft Windows on a personal computer.

### Ethical considerations

Ethical clearance was obtained from the Sefako Makgatho Health Sciences University Research Ethics Committee (SMUREC) prior to commencing with the study (ref. no. SMUREC/M/13/2018:PG, 2018).

## Results

Forty-four black male patients with congenital haemophilia were managed at DGMAH during the study period. The age of patients with congenital haemophilia in this study ranged from eight days to 52 years, with a mean age of 18.46 years (see Table 1).

**TABLE 1:** Summary of demographics of patients with congenital haemophilia at Dr George Mukhari Academic Hospital.

Study population (*n* = 44)	Frequency	Percentage
**Gender**		
Male	44	100.00
Female	0	0.00
**Age group in years**		
0–12	17	38.64
13–19	8	18.18
20–35	13	29.55
36–50	5	11.36
> 50	1	2.27

**FIGURE 1:**
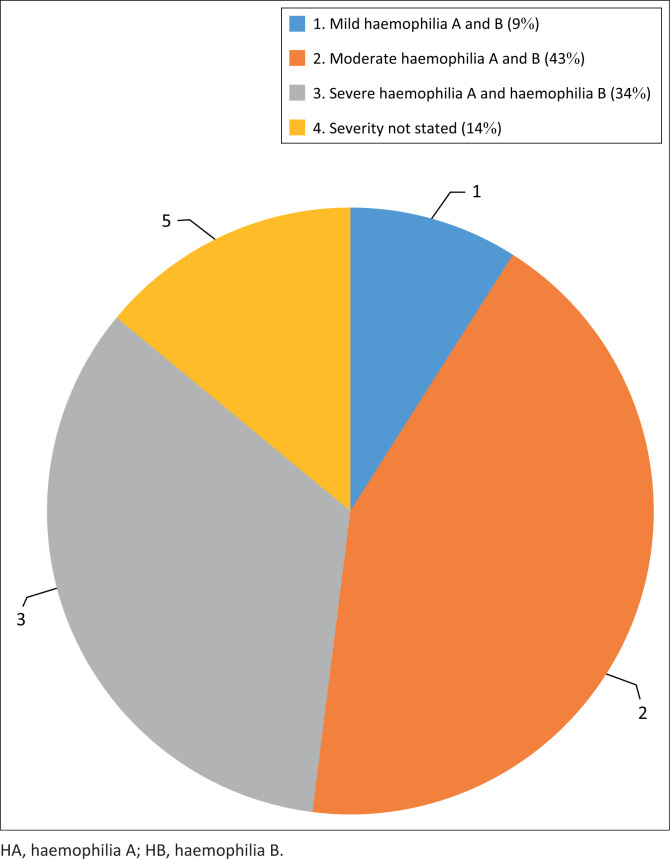
Distribution of disease severity amongst all patients with congenital haemophilia.

Haemophilia A was more prevalent (81.82%) than haemophilia B (18.18%). Moderate HA comprised 47%, followed by severe HA at 31% and mild HA at 11%. Severe HB was more common at 50%, compared with moderate HB at 25%. In approximately 14% of cases, the severity was not stated (Table 2 and Figure 1).

**TABLE 2:** Haemophilia versus disease severity by factor assay levels.

Type of haemophilia	Disease severity			
	Mild	Moderate	Severe	Not stated
**Haemophilia**				
A				
*n* (36)	4	17	11	4
% of total	11	47	31	11
B				
*n* (8)	0	2	4	2
% of total	0.0	25	50	25
**Total**				
HA & HB				
*n* (44)	4	19	15	6
% of Total	9	43	34	14

Note: *n* = number of PWH.

HA, haemophilia A; HB, haemophilia B; PWH, patients with haemophilia.

**FIGURE 2:**
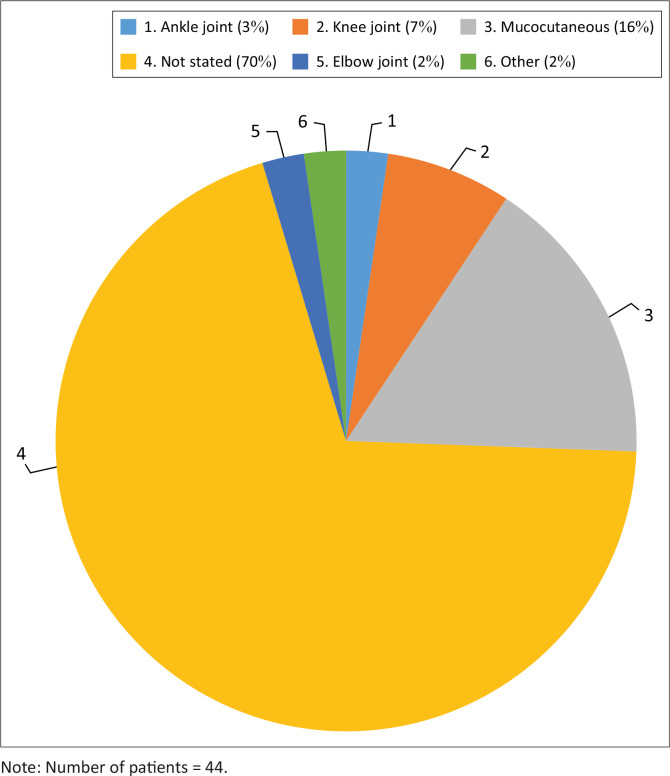
Bleeding sites in patients with haemophilia at Dr George Mukhari Academic Hospital.

The most frequently documented clinical presentation was spontaneous mucocutaneous bleeding, followed by joint bleeds (Figure 2). The knee was the most commonly affected joint. The bleeding site was not stated at presentation and at diagnosis in 70% of PWH in this study. Spontaneous mucocutanoeus bleeding included epistaxis and gum bleeding at 11% and 3%, respectively. Other spontaneous bleeding sites included the scrotum in 2% of cases. Spontaneous bleeding was reported at presentation to DGMAH, at diagnosis and at follow-up visits. Records of bleeding in more than one site were mostly trauma related and were categorised as induced bleeding. These induced bleeding episodes included tooth extraction, lip injury, circumcision, head injury, wrist and knee injuries.

A positive family history of haemophilia was observed in 50% of the patients, including maternal uncles (31.82%) and male siblings (18.18%). In approximately half of the patients (49%), the presence or absence of family history was not stated in patient records. Screening tests and genetic testing were not performed to detect haemophilia carrier status.

An isolated prolonged APTT with a normal prothrombin time (PT)/International Normalised Ratio (INR), normal platelet count and fibrinogen were found in all PWH. The diagnosis was confirmed with one-stage factor assays, and genetic testing was not routinely performed. Absence of genetic records precluded genetic risk stratification of patients for inhibitor development in this study population. Patient records did not reflect routine testing results for family members. Of note, in 11% of PWH, the diagnosis was established 3–10 years after their first bleeding, which included mucocutaneous and joint bleeds.

Disease-related complications included joint complications and life-threatening bleeding. Joints included the knees (7%), ankles (2%) and elbows (2%) in patients with moderate and severe haemophilia. This study revealed that joint complications were less prevalent in severe HA (2%) when compared with moderate HA (9%). Patients with mild haemophiliacs experienced no joint complications. Target joints were reported only in moderate HA at DGMAH. Life-threatening bleeding comprised 4% of all cases and included massive gastro-intestinal and retropharyngeal bleeds.

Inhibitor development was seen in 18% of patients with moderate and severe HA, and none of these patients had a family history of inhibitor development. No inhibitors were reported in patients with HB in this study. Review of records did not reveal hepatitis A, B, C or human immunodeficiency virus (HIV) seroconversion in any of the PWH. These patients were tested annually for hepatitis and biannually for inhibitor screening, as per South African treatment guidelines for haemophilia.

Inhibitor development documented in 18% of the cases included high-titre inhibitors, that is, > 5 Bethesda units (BU) in 11% of patients with severe HA and low-titre inhibitors (< 5 BU) in 7% of patients with moderate HA (Table 3). The association between inhibitor development and disease severity was statistically significant (*p* < 0.05).

**TABLE 3:** The profile of inhibitor development.

Inhibitor description	Disease severity				
	Mild	Moderate	Severe	Not stated	Total
Number of patients	4	16	10	6	36
**Inhibitor titre**					
< 5 BU	0	3	0	0	3
> 5 BU	0	0	5	0	5

**Total**	**4**	**19**	**15**	**6**	**44**

BU, Bethesda unit.

A single female patient was recorded to have acquired haemophilia. She was 61 years old and presented at the age of 56 years with epistaxis for a duration of six months and joint stiffness which had been longstanding. She had neither a previous history of a bleeding diathesis nor a family history of bleeding. She reported a history of rheumatoid arthritis for a duration of five years and admitted to having defaulted treatment. Physical examination revealed joint deformities, including the swan neck deformity in the fifth digit of her right hand and a bilateral hallux valgus. The full blood count and biochemistry showed an iron deficiency anaemia. Coagulation screening revealed an isolated prolonged APTT, which failed to correct with mixing studies. The one-stage factor assay revealed a FVIII activity level of 38%, and the inhibitor titre was 9 BU. A diagnosis of acquired HA was made.

Management of PWH at DGMAH adopts a multidisciplinary approach, as advocated by the WFH guidelines.^11^ Medical intervention included episodic plasma-derived FRT, adjuvant therapy including tranexamic acid, analgesia and vaccinations. Medical records did not reflect the use of prophylaxis. There was a paucity of documented data regarding psychosocial support, physiotherapy and genetic counselling.

In PWH without or with low-titre inhibitors (< 5 BU), medical intervention included episodic FRT with plasma-derived, solvent detergent-treated Haemosolvate^®^ Factor VIII (National Bioproducts Institute) and factor IX complex product Haemosolvex^®^ (National Bioproducts Institute) for patients with HA and HB, respectively. Factor VIII inhibitor bypassing agent (FEIBA), an activated prothrombin complex concentrate (Adcock Ingram Critical Care), was used to manage PWH with high-titre inhibitors (> 5 BU) and bleeding. Inhibitor eradication protocol was not available during the study period. Medical records did not reflect the use of a recombinant FVIIa concentrate, NovoSeven^®^ (Novo Nordisk), in these patients. At the time of the study, only 13% of patients were on home therapy. Home therapy was introduced at DGMAH in February 2017. No patients were recorded to be on prophylaxis. Adjuvant therapy included tranexamic acid and analgesic agents (paracetamol and opioids). Other recorded interventions in patients with moderate and severe haemophilia included physiotherapy (25%) and social grants (13%). Psychological support, genetic counselling and family planning were not documented throughout the observation period.

## Discussion

In South Africa, the health needs of the population exceed the capacity of the health care system; therefore, the medical care of PWH is often not afforded the same level of urgency as many of the other national health care priorities.^1^ The data from this study highlights the importance of haemophilia awareness amongst health care professionals in order to reduce time to diagnosis and the need for electronic record-keeping when managing patients with long-term needs for medical care.

The results of this study confirm that HA was more common (82%) than HB (18%) at DGMAH, which is comparable to what has been reported both locally and internationally.^14,16^Haemophilia has no predilection for race or geographic area; thus, all ethnic groups can be affected.^2,13^ In this study, all PWH were of black ethnicity, which is expected as DGMAH is situated in a geographic area populated mostly by this ethnic group. As haemophilia is an X-linked recessive disorder, it was not surprising that the patients were predominantly male. Females are usually carriers and are often asymptomatic,^11,13^ but they can present with bleeding symptoms if compound heterozygosity, skewed lyonization or X chromosome loss is present.^17,18^ The one female patient in the study population, known to have rheumatoid arthritis, was diagnosed with acquired HA. Acquired HA is a rare, potentially life-threatening autoimmune condition that is seen in ~1 per million of the population per year.^19^ It may affect both sexes and is more frequently seen in the elderly. The majority of cases are idiopathic; however, it has been associated with pregnancy, infections, drugs, auto-immune diseases (most commonly rheumatoid arthritis) and malignancy. The bleeding in acquired HA occurs as a result of the formation of autoantibodies against endogenous FVIII, and in contrast to congenital haemophilia, subcutaneous bleeds are most common.^19^

The mean age of 18.46 years was comparable to the 16.2 years reported in a study performed in Cameroon.^9^ Lack of awareness of the disease as well as availability of diagnostic facilities has possibly contributed to delay in diagnosis, as documented in studies performed in South Africa and Cameroon.^8,9^ Bleeding symptoms in severe haemophilia have been described to include spontaneous joint and muscle bleeds, with mucocutaneous bleeds being the least common.^7^ In contrast, at DGMAH, mucocutaneous bleeds were the most frequent site recorded in severe haemophilia, but this result may be skewed as the bleeding site was not recorded in 70% of PWH. In this study, spontaneous bleeding symptoms were reported more commonly in patients with moderate HA throughout the follow-up period as compared with severe HA, whereas in patients with HB, spontaneous bleeding was seen more frequently in severe HB.

Approximately 70.0% of PWH have a positive family history, which may be absent in a third of patients, where it is attributed to spontaneous mutation.^13,14^ This prevalence is comparable to what has been reported in India (64.13%);^20^ however, at DGMAH, the prevalence of a family history was slightly less at 50.0%, which is more than the study conducted in the Western Cape that reported a prevalence of 38.0%.^8^ A recognised, serious complication of haemophilia is the development of anti-factor antibodies, which requires medical expertise, diagnostic facilities and anti-inhibitor treatment for appropriate management. Risk factors for developing inhibitors in patients receiving FRT includes positive family history and black ethnicity.^21,22^ Patients with inhibitors at DGMAH had no reported positive family history for inhibitor development, in contrast to Mafika et al., where a positive family history was present in 60.0% of patients with inhibitors.^23^

Genotyping is considered superior in predicting inhibitor development,^24,25^ but the lack of genetic records precluded risk stratification of PWH at DGMAH. Genetic counselling and genotyping should be incorporated into the standard of care for PWH.^11^

The stratification of patients according to prevalence of disease severity in this study (that is, moderate HA, followed by severe and mild disease) differs from the results of the study by Mahlangu et al.,^1^ where they reported the majority of PWH to have severe HA (68%), followed by mild (17%) and moderate (15%) disease. The stratification for HB according to prevalence of disease severity was comparable for severe HB (50% vs 49%) to that reported by Mahlangu et al.;^1^ however, mild HB disease was the next most common form at 30%, compared with moderate HB at 22%. At DGMAH, there were no recorded cases of mild HB.

In this study, patients with severe and moderate HA and HB presented earlier with spontaneous bleeds compared with PWH with mild disease, which is similar to what is reported in literature.^13^

It was observed that moderate HA patients had more spontaneous bleeds and joint complications when compared with severe HA, but these data need to be interpreted with caution, as the clinical information in 41% of patients was incomplete, that is, 7 severe HA, 8 moderate HA and 3 mild HA. It has been reported that the clinical phenotype may improve with age, depending on mutation, and therefore some patients with severe HA may present with a mild bleeding phenotype.^7^ In the literature, variation in clinical phenotype has been attributed to co-inheritance of inherited thrombophilia such as deficiencies in protein C, protein S, antithrombin and factor V Leiden mutation.^7^

Joint complications are commonly associated with severe HA, and this is attributed to recurrent bleeds which trigger an inflammatory process in the synovium.^26,27^ In literature, knee joints are the most affected, similar to what was found in this study.^16^ In this study patients with moderate haemophilia had more joint complications compared to those with severe haemophilia which differs from what is reported in literature, where joint complication is considered to be more common in severe haemophilia compared with moderate haemophilia.^28^ The LTB was infrequent in this study and was associated with spontaneous massive gastrointestinal tract and retropharyngeal bleeds in severe haemophilia. This is comparable to what has been reported in literature, where LTB comprises 1% – 4% of cases of bleeds in severe haemophilia.^10,11^

Home therapy was introduced in February 2017, and the uptake of 13% is lower compared with what has been reported internationally (90%)^29^; however, a survey performed in SA revealed 0% – 70% uptake.^30^ Home therapy is beneficial in reducing joint pain, joint destruction and disability and hospitalisation for disease complications.^29,30^ The most frequently reported treatment-related complication in this study was development of inhibitors. The prevalence of inhibitors is reported to be 20% – 40% in HA, which is comparable to the findings of 18% at DGMAH.^31^ Eleven per cent of these patients had high-titre inhibitors and were treated with FEIBA, with no records of recombinant FVIIa use. The reported prevalence of inhibitors in patients with HB is low at 3%.^31^

Immune tolerance induction was not documented to be the standard of care in the treatment of haemophilia patients with high-titre inhibitors at DGMAH.

## Conclusion

**FIGURE 3:**
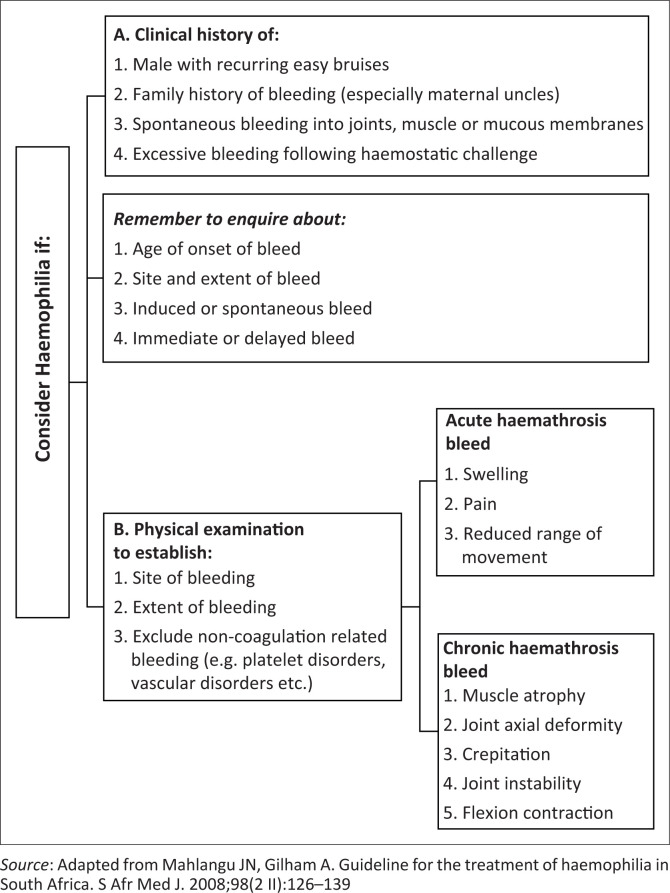
Algorithm to guide the clinical approach to a patient with suspected haemophilia.

**FIGURE 4:**
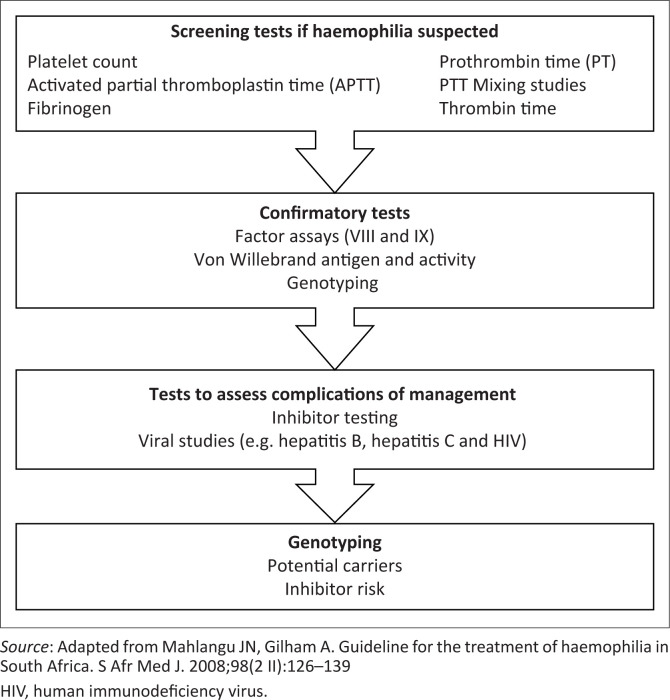
Laboratory approach to a patient suspected of having haemophilia.

Genotyping will be useful in confirming the diagnosis, assisting in risk stratification for development of inhibitors and identification of carriers. Expanding the home therapy and prophylaxis programmes will likely improve the quality of life in our patients. The review of records suggests that the multidisciplinary approach (including physiotherapy, psychosocial and genetic counselling) requires strengthening. Record-keeping was recognised to be a major limitation in this study. Electronic record-keeping by treaters and encouraging the use of bleeding charts by patients could help to address this challenge. In order to improve haemophilia care in our setting, we recommend the use of the following diagnostic algorithms derived from the South African and WFH guidelines^11,14^ (Figures 3 and 4).^14^
